# Cystitis in Women*Read before the Bar Harbor Hospital Club, June 28, 1899.

**Published:** 1899-09

**Authors:** 


### CYSTITIS IN WOMEN.*

### The Importance of Local Treatment.

Inflammation of the bladder is more frequent among women than among men, because in women many more causes operate to bring about the disease. Among the causes not common to men may be mentioned the short urethral canal, allowing a more ready extension of inflammation; the position of the uterus and its adnexæ immediately behind the posterior wall of the bladder, the various inflammatory diseases occurring in these organs and the tumors arising therefrom; the adhesions which frequently remain after attacks of local pelvic peritonitis, limiting the free dilatation and contraction of the bladder walls. Other causes are the various lacerations occurring in consequence of labor. These vaginal and perineal lacerations allowing procidentia and prolapse of the vaginal walls favor a dislocation of the bladder which causes the base of this organ to occupy a lower plane than the internal orifice of the urethra. As a result we find that the bladder never empties itself entirely, so that there always remains a small quantity of so-called residual urine, which, quickly undergoing decomposition and containing much sediment and debris, lights up a troublesome cystitis.

A prolonged and tedious labor frequently results in a cystitis by reason of long pressure of the child's head upon the bladder, paralyzing its walls or causing a low grade pressure necrosis involving the three walls of the bladder, and causing a slough which produces a vesico-vaginal fistula. Happily this unfortunate accident is uncommon in these days of improved obstetrical practice. Another frequent cause of cystitis is pressure of the child's head upon the full bladder during the third stage of labor. This is a cause which is

*Read before the Bar Harbor Hospital Club, June 28, 1899.

rarely referred to by writers, and yet it accounts for many cases of paralysis of the bladder after labor; cases requiring prolonged catheterization before the muscular walls of the bladder regain their tone. Nine-tenths of these cases result in cystitis owing to infection from imperfect technique and carelessness in catheterization. In this connection it is well to emphasize the importance of seeing that the bladder is emptied before the third stage of labor begins. If the patient cannot accomplish this herself, she should be carefully catheterized. Other common causes of cystitis are gonorrhœa, latent gonorrhœa, tuberculosis, exposure to cold, injury from coitus, retention of urine, and stone in the bladder.

As to the pathology, it suffices for the purpose of this article to say that it is the same as that of inflammation of the mucous membrane in other parts of the body. We have the acute, the subacute and chronic forms of inflammation, and the process may be either local or diffuse. The inflammation may extend beyond the epithelium and the submucosa, and involve the muscular walls and even the peritoneum. It may even extend beyond and involve the connective tissue near.

The treatment of these various conditions is quite simple and satisfactory if properly understood. The advice usually laid down in the books is misleading and not sufficiently positive. I wish to emphasize very positively the opinion that some form of local treatment is indicated in almost all cases of cystitis occurring in women. Of course the old maxim to avoid local treatment in the first stages of an acute cystitis is to be considered, but even in these cases, I hold that if the symptoms do not yield to the usual constitutional measures in a very few days, one should not hesitate to begin local treatment.

Much benefit and great relief will result from a careful lavage of the bladder with any one of the mild cleansing solutions. The washing should be done under strict antiseptic precautions, care being taken not to overdistend the bladder. A very simple apparatus for the purpose is here shown. A pint jar with an opening at the bottom having a rubber tube attached and a two way catheter at the end of the tube, is filled with the desired solution. This may be a deci-normal salt solution; or a solution of boracic acid one ounce to Oi. of water, or in cases of gonorrhœal cystitis, a solution of nitrate of silver $\frac{1}{2}$ to 1 or 2 grains to the ounce. After the urine has been carefully drawn, a small amount of the solution, one to four ounces, is allowed to flow into the bladder. This is drawn off or allowed to flow off, and the process is repeated until the water comes away clear.

In this manner an enormous amount of debris, shreds of mucus and exfoliated epithelium can be washed out of the bladder. This proceeding, depending upon the severity of the case, can be carried out from one to three times a day. The patient should be confined absolutely to bed; the diet should be of the blandest kind (if possible a milk diet should be insisted upon) and large quantities of diluent drinks should be prescribed. Other measures, depending upon the cause, may be necessary, but as a rule the above treatment will be found efficient in most cases of acute cystitis.

The majority of cases of cystitis in women are of the subacute or chronic varieties. Depending as they do upon such a variety of causes, these cases require many forms of local treatment. By local treatment in this connection I do not mean direct application to the bladder walls, but treatment directed towards correcting the cause which has produced the cystitis. Uterine deviations and misplacements must be corrected, diseases of the ovaries and tubes and pelvic adhesions must be overcome, lacerations must be repaired, calculi removed, etc.

In addition to lavage of the bladder, we have another method of local treatment. This is the treatment by direct applications to the bladder walls through the endoscope. It is especially valuable in those cases where the disease is localized in one or more spots or areas in the bladder. The patient being placed in the knee-chest or in an elevated dorsal position, the endoscope is introduced, and the bladder, having previously been emptied, fills at once with air. Its entire surface can then be carefully inspected through the endoscope by means of the headlight. Spots of local inflammation can thus be treated by applications of much stronger solutions that can be used as a wash. Often one or two applications are sufficient for the cure of cases which have long resisted all other forms of treatment. The most satisfactory solutions for most cases, especially of gonorrhœa, tuberculosis and socalled solitary ulcer of the bladder, are the solutions of nitrate of silver from five to twenty grains to the ounce. Occasionally much stronger solutions may be used, if applied with skill and the greatest care.

As a last resort in those cases of cystitis which do not yield to the measures indicated above, we are justified in establishing a vesico-vaginal fistula, to furnish an opening for the continuous flow of urine, and to put the bladder at rest.

The patient is allowed to be about, wearing a large pad of some absorbent material to take up the urine. The pad should be constantly changed and the parts kept scrupulously clean. This is best accompanied by frequent washing and the use of thick zinc ointment. At the end of a few months the mucous membrane of the bladder is restored to a healthy condition, and the fistula may be closed.—*Journal of Medicine and Science, Maine.*

